# Automating quality control in cardiac magnetic resonance: Artificial intelligence for discriminative assessment of planning and motion artifacts and real-time reacquisition guidance

**DOI:** 10.1016/j.jocmr.2024.101067

**Published:** 2024-07-28

**Authors:** Hoi C. Cheung, Kavitha Vimalesvaran, Sameer Zaman, Michalis Michaelides, Matthew J. Shun-Shin, Darrel P. Francis, Graham D. Cole, James P. Howard

**Affiliations:** National Heart and Lung Institute, Imperial College London, London, United Kingdom

**Keywords:** Artificial intelligence, Machine learning, Cardiac magnetic resonance, Quality control, Quality assessment, Convolutional neural networks

## Abstract

**Background:**

Accurate measurements from cardiovascular magnetic resonance (CMR) images require precise positioning of scan planes and elimination of motion artifacts from arrhythmia or breathing. Unidentified or incorrectly managed artifacts degrade image quality, invalidate clinical measurements, and decrease diagnostic confidence. Currently, radiographers must manually inspect each acquired image to confirm diagnostic quality and decide whether reacquisition or a change in sequences is warranted. We aimed to develop artificial intelligence (AI) to provide continuous quality scores across different quality domains, and from these, determine whether cines are clinically adequate, require replanning, or warrant a change in protocol.

**Methods:**

A three-dimensional convolutional neural network was trained to predict cine quality graded on a continuous scale by a level 3 CMR expert, focusing separately on planning and motion artifacts. It incorporated four distinct output heads for the assessment of image quality in terms of (a, b, c) 2-, 3- and 4-chamber misplanning, and (d) long- and short-axis arrhythmia/breathing artifact. Backpropagation was selectively performed across these heads based on the labels present for each cine. Each image in the testing set was reported by four level 3 CMR experts, providing a consensus on clinical adequacy. The AI's assessment of image quality and ability to identify images requiring replanning or sequence changes were evaluated with Spearman’s rho and the area under receiver operating characteristic curve (AUROC), respectively.

**Results:**

A total of 1940 cines across 1387 studies were included. On the test set of 383 cines, AI-judged image quality correlated strongly with expert judgment, with Spearman’s rho of 0.84, 0.84, 0.81, and 0.81 for 2-, 3- and 4-chamber planning quality and the extent of arrhythmia or breathing artifacts, respectively. The AI also showed high efficacy in flagging clinically inadequate cines (AUROC 0.88, 0.93, and 0.93 for identifying misplanning of 2-, 3- and 4-chamber cines, and 0.90 for identifying movement artifacts).

**Conclusion:**

AI can assess distinct domains of CMR cine quality and provide continuous quality scores that correlate closely with a consensus of experts. These ratings could be used to identify cases where reacquisition is warranted and guide corrective actions to optimize image quality, including replanning, prospective gating, or real-time imaging.

## Graphical abstract

**Figure:**
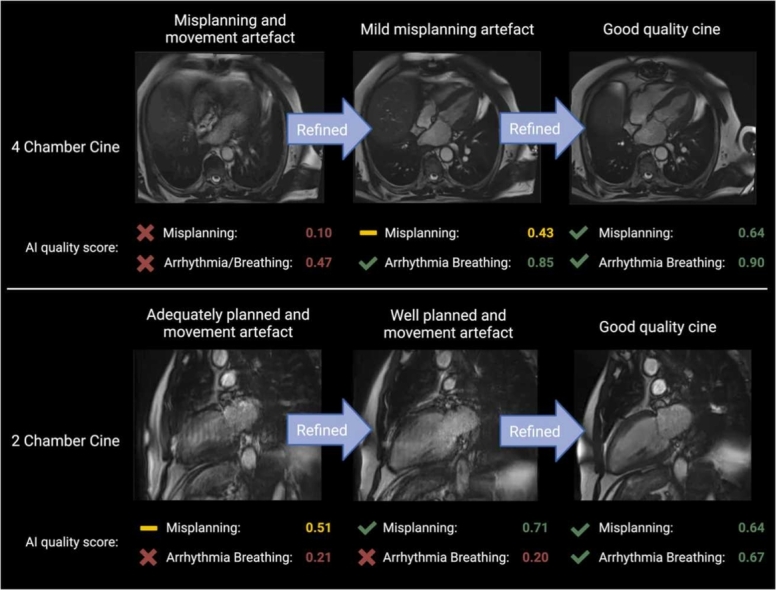

## 1 Background

Cardiovascular magnetic resonance (CMR) is the gold standard for the assessment of cardiac structure and function [1]. CMR measurements are used by doctors to decide on drug and device therapies in not only heart failure but also other fields, such as oncology [2]. Tremendous efforts have therefore been made to develop artificial intelligence (AI) to improve the precision with which we can estimate cardiac mass [3], ejection fraction [4], and longitudinal strain [5].

All of these AI approaches, however, are contingent on having high-quality images to analyze. For example, foreshortening in long-axis (LAX) cine imaging falsely increases left ventricular (LV) strain, reduces biplanar ventricular volumes, and increases biplanar ejection fraction [6]. Images with motion artifact (e.g. due to breathing) make accurate delineation of cardiac structures difficult, and, particularly in the case of arrhythmias, sometimes inappropriate to even make measurements [7].

Despite this, comparatively little effort has been devoted to systematic quality control. Radiographers must currently manually inspect each acquired image to confirm diagnostic quality and decide whether reacquisition is warranted. Furthermore, acquisition will only be successful if the underlying cause is identified; motion artifacts may be resolved by real-time imaging, whereas a misaligned image will require replanning [8].

In this study, we explore the ability of AI to automatically grade the quality of LAX and short-axis (SAX) CMR cine imaging, by grading them on a continuous scale for both planning quality and movement artifacts. We then investigate the AI’s ability to identify whether cines are clinically adequate or require flagging to radiographers for reacquisition. Finally, we implement the trained AI as a working prototype at our institution and demonstrate it performing real-time quality control.

## 2 Methods

### 2.1 Study design

An AI was trained to assess cine imaging quality across the separate domains of planning (slice positioning) and freedom from movement artifact (due to arrhythmia or breathing). Identifying whether arrhythmia or breathing is present in a cine could be argued to be relatively view agnostic, as similar artifacts are introduced regardless of the scan plane. However, to decide on whether a cine is well-planned requires us to know what the radiographer had aimed to capture; a 3-chamber cine should include the LV outflow tract, whereas a 4-chamber cine should not. For this reason, the problem was framed as four separate tasks. For each input cine, the AI assesses planning quality if the image had been intended as a (a) 2-, (b) 3- and (c) 4-chamber LAX image, and the AI also provides (d) the amount of movement artifact present. The AI was not trained to assess SAX planning quality (only motion artifact); the SAX stack is relatively robust to misplanning due to its slice-wise analysis.

The study therefore comprises four subdatasets: 2-, 3- and 4-chamber misplanning datasets and a motion artifact dataset (including all LAX and SAX views).

### 2.2 Source data

**Fig. 1:**
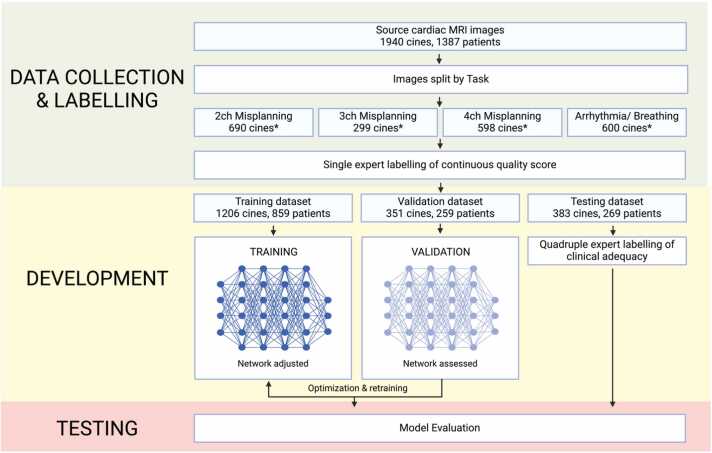
The study was executed in three phases: data collection and labeling, development, and testing. In the development phase, the neural network was adjusted with training data. The validation data were used to gauge the performance of the network on unseen data during this process so that optimal training settings (hyperparameters) could be explored. After the final model was trained, it was finally evaluated on the hold-out testing set. *Note the total number of cines is less than the sum of cines across the tasks, as a single cine could contribute toward both a misplanning dataset and an arrhythmia or breathing dataset. MRI: magnetic resonance imaging.

CMR studies were extracted from the Imperial College Healthcare National Health Service (NHS) Trust database in Digital Imaging and Communications in Medicine (DICOM) format. Ethical approval was granted by the Health Research Authority (Integrated Research Application System number 309103). Studies were obtained from two Siemens (Siemens Healthineers, Munich, Germany) Area 1.5T scanners and one GE (GE Healthcare, Chicago, Illinois, ) SIGNA 1.5T scanner. To enrich the dataset for artifacts, we identified studies where multiple cine images of the same series description were repeated, with the rationale that instances exhibiting artifacts or of suboptimal quality often necessitate reacquisition. Two-, 3- and 4-chamber LAX and SAX cine imaging were included. Patients were randomized in a 3:1:1 ratio to the training, validation, and testing datasets (Fig. 1).

### 2.3 Data labeling

The associated video loops of the studies were manually evaluated using the Unity Imaging software developed by the Unity Imaging Collaborative Howard et al. [9].

The problem was posed as four individual tasks for the AI, with their own training, validation, and testing datasets (see Section 2.1); misplanning artifacts were evaluated for LAX cines according to the view (2-, 3-, or 4-chamber). The misplanning task was not applied to SAX sections as planning these images is relatively trivial. Conversely, arrhythmia or breathing artifacts are global features and therefore were evaluated collectively across all LAX and SAX sections.

The training, validation, and testing datasets were graded on a continuous scale for image quality by a senior cardiologist (J.P.H.) with level 3 CMR certification and normalized to values between 0 and 1. This was accomplished by an algorithm written by the author J.P.H., based on the Glicko-2 chess rating system [10] and published previously by our group [11]. Over 10,000 comparisons were performed for each task.

Four level 3 certified CMR physicians (G.D.C., J.P.H., K.V., and S.Z.) were then asked to judge whether each cine in the testing dataset was either diagnostically adequate or inadequate (and therefore they would want the cine reacquired).

These labels were used to derive consensus labels, where a non-diagnostic label was assigned when at least two experts judged the case as non-diagnostic and needing reacquisition.

### 2.4 Neural network design and training

We used a form of AI called a convolutional neural network, which is inspired by the mammalian visual cortex and excels at processing image data [12]. An Inflated 3D ResNet-50 model architecture was used with the PyTorch machine learning framework [13]. It is a three-dimensional (3D) convolutional neural network that inflates the standard two-dimensional (2D) layers of ResNet-50 [14] into 3D for spatiotemporal feature learning. We modified the model to have four output layers (2-chamber misplanning, 3-chamber misplanning, 4-chamber misplanning, and 2-chamber/3-chamber/4-chamber/SAX arrhythmia or breathing). A sigmoid activation function was used after each output to scale the predictions between 0 and 1 (0 being the worst quality image within the datasets and 1 being the best). The task was modeled as a regression problem. Loss was calculated using mean squared error [15], [16] over a batch size of 16 for 50 epochs and reduced using the AdamW optimizer, with a weight decay of 5.e0-5. The learning rate began at 1.0e-4 and was increased up to 5.0e-4 and then decayed using a OneCycle learning rate scheduler.

The original DICOMs of the labeled video loops were extracted and resized to 256 by 256 during training using random resized cropping with a scale range of 60% to 100%. Images were resized to 320 by 320 during training and validation to reduce the “train-test discrepancy” [17]. Pixel values were normalized to be between 0 and 1.

Frame-wise augmentations were performed using the Albumentations library [18]. Augmentations were randomly selected at the video level with a probability of 35% for each being applied; these were random flipping, Gaussian and motion blurring, Gaussian and multiplicative noise, random shifting, scaling and rotating, optical or grid distortion, random brightness and contrast, random sharpening, and random resized cropping. These augmentations were selected through hyperparameter sweeping. Each frame within a video was augmented identically to ensure only spatial augmentation occurs.

Performance on the validation dataset was used to fine-tune model hyperparameters and assess model performance and generalizability before testing with unseen data.

We used a single network trained to perform all four tasks as we found this performed better than training four separate networks trained on the four subsets of the total dataset (Supplementary Appendix Table A1). This required a training approach that could allow for cines in the dataset to be labeled for only one task (either judged in terms of its planning quality for its own view, or freedom from movement artifact, or both). For non-labeled tasks, dummy labels were used and all gradients from these outputs were excluded from backpropagation by not contributing to the final loss. In addition, because each view was only labeled for planning quality in terms of its own view (e.g. a 4-chamber view was only assessed for how good a 4-chamber image it was), labels were not present for the other views. One approach would be to assume an image quality score of 0 for the other views (i.e. a 4-chamber cine must be of minimum quality when judged as a 2- or 3-chamber), but this is naive; the most misplanned images contain no heart altogether, and some 4-chamber images may actually be inadvertently approaching a 3-chamber, and therefore assuming a “terrible” label of 0 would be inappropriate. We therefore used an approach of selective backpropagation, where backpropagation was only performed against a dummy label of 0 (minimal quality) when the AI predicted a quality score ≥0.5 for a view not present. This trained the AI to predict low-quality scores for the non-present views for each cine, without being forced to predict exactly 0 for these views. Supplementary Appendix Fig. A1 shows the resultant impact of this approach on the distribution of quality scores for non-present views. The value of 0.5 was chosen through a hyperparameter sweep with reference to the validation dataset.

### 2.5 Statistical analysis

The neural network was trained to assess image quality in terms of planning and freedom from motion artifact. This was assessed using Spearman’s rank correlation coefficient (rho) between the AI’s predictions on the testing dataset and the expert labels. This metric was prespecified as we predicted quality scores were at risk of being non-normally (bimodally) distributed with unequal variance if a large proportion of cases were completely artifact-free, and a discrete separate population of low-quality images existed. This would preclude more traditional measures, such as intraclass correlation coefficient and R-squared.

We also assessed the ability of the network to function as a binary classifier that identifies whether an image is diagnostically adequate or inadequate (and therefore needs repeating). Receiver operating characteristic (ROC) curves were plotted for each of the four tasks (see Section 2.1), where the input was the AI’s assessment of image quality and the gold standard was the experts' consensus across the four experts (see Section 2.3). Areas under ROC (AUROC) were calculated with their 95% confidence intervals (CI) using the pROC package [19]. We also report sensitivity and specificity for these tasks at the cutoff defined by Youden’s Index. Statistical analysis was performed using the R programming language [20], version 2023.12.0+369.

### 2.6 Implementation

A proof of concept implementation was introduced following the testing process on a Siemens 1.5T Aera scanner running the syngo E11C operating system and the prototype Siemens Framework for Image Reconstruction Environments (FIRE) [21]. The trained neural network was exported in the Onnx format and inference was performed using a workstation with a 4 Quadro P4000 Graphics Processing Units (Nvidia, Santa Clara, California). The workflow was designed to return an additional image to the scanner along with the acquired cine, which contained information across the two quality domains (planning quality and extent of arrhythmia or breathing artifacts) and a decision on whether the image was clinically adequate.

## 3 Results

A total of 1940 cines across 1387 studies (1387 patients) were included (Table 1; Fig. 1). [A total of] 1206, 351, and 383 cines were assigned to the training, validation, and testing sets, respectively. Exactly 59.1% (820/1387) of patients were male, the mean age was 58.2 years old, and 94.8% (1315/1387) of scans were from Siemens machines (Table 2). The clinical indications for the scans are shown in Supplementary Appendix Table A2—of the studies with available imaging reports, 5.1% (58/1142) were performed for pulmonary hypertension and 1.9% (22/1142) for congenital heart disease.

**Table 1:** Distribution of cines and their corresponding studies across different labels (2-chamber misplanning, 3-chamber misplanning, 4-chamber misplanning, and arrhythmia or breathing) for training, validation, and testing datasets.

	Training	Validation	Testing	Total
Studies	859	259	269	1387
Cines	1206*	351*	383*	1940*
2-chamber misplanning cines	441	121	128	690
3-chamber misplanning cines	171	57	71	299
4-chamber misplanning cines	379	109	110	598
Arrhythmia or breathing cines (2-/3-/4-chamber and short-axis)	367	115	118	600

* Note the total number of cines is less than the sum of cines across the tasks, as a single cine could contribute toward both a misplanning dataset and an arrhythmia or breathing dataset.

**Table 2:** Patient and scanner characteristics across the training, validation, and testing datasets.

		Training	Validation	Testing	Total
Patient characteristics		859	259	269	1387
Sex	M	502 (58.4%)	155 (59.8%)	163 (60.6%)	820 (59.1%)
	F	357 (41.6%)	104 (40.2%)	106 (39.4%)	567 (40.9%)
Mean age		58.1 ± 16.4	57.9 ± 16.6	58.9 ± 16.8	58.2 ± 16.5
Manufacturer	Siemens	810 (94.3%)	244 (94.2%)	261 (97.0%)	1315 (94.8%)
	GE	49 (5.7%)	15 (5.8%)	8 (3.0%)	72 (5.2%)

Counts are numbers and percentages; continuous measures are numbers and standard deviations.

### 3.1 Quality assessment of long-axis image planning

A total of 309 cine images (110 4-chamber, 128 2-chamber, and 71 3-chamber) from 227 unique studies were assigned to the testing dataset and were used to assess the AI’s ability to continuously judge image quality in terms of slice planning.

The AI’s assessments were compared with the expert-derived quality assessments.

**Fig. 2:**
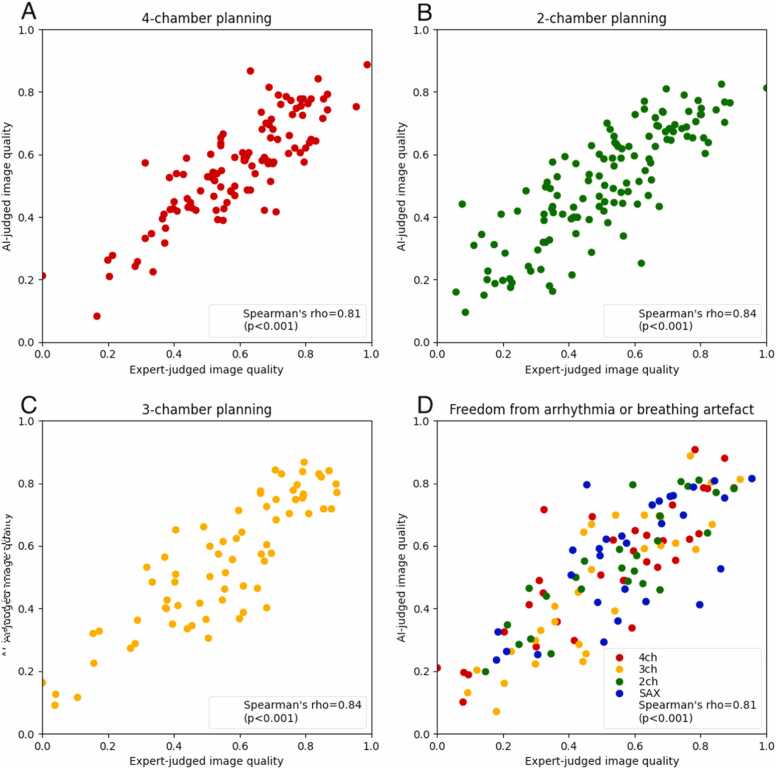
Correlation between AI-judged and expert-judged image quality on the testing set. Spearman’s rank correlation between AI and expert assessments of image quality (A: 4-chamber planning; B: 2-chamber planning; C: 3-chamber planning; D: freedom from motion artifact across all four views). *AI* artificial intelligence, *2Ch* 2-chamber, *3Ch* 3-chamber, *4Ch* 4-chamber, *SAX* short-axis.

For 2-, 3- and 4-chamber LAX cines, Spearman’s rho was 0.84 (p < 0.001), 0.84 (p < 0.001), and 0.81 (p < 0.001), respectively (Fig. 2A-C, respectively).

Across the testing dataset, the 4 experts deemed 66 of 309 cines (21%) as diagnostically inadequate (and needing repeating) in terms of slice planning (20 of 128 (16%) 2-chamber cines, 15 of 71 (21%) 3-chamber cines, and 31 of 110 (28%) 4-chamber cines).

**Fig. 3:**
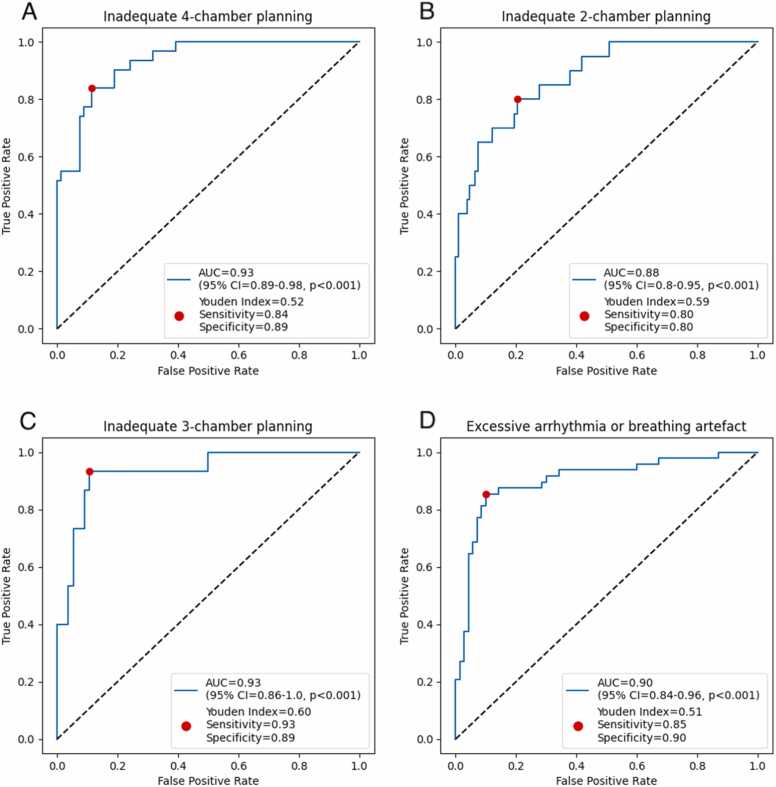
Ability of AI to identify cines judged clinically inadequate and requiring repeating on the testing set. Receiver operating characteristics curves for the AI in identifying cines that are clinically inadequate and therefore require repeating. Youden Indices and corresponding sensitivities and specificities are also illustrated (A: inadequate 4-chamber planning; B: inadequate 2-chamber planning; C: inadequate 3-chamber planning; and D: excessive movement artifact across all 4 views). *AUC* area under the curve, *CI* confidence interval, *AI* artificial intelligence.

By providing continuous ratings of image quality and applying a cutoff to these, the AI can be used to identify inadequately planned cines that require repeating to ensure diagnostic quality: for 2-chamber images, the AUROC was 0.88 (95% CI 0.80 to 0.95, p < 0.001), sensitivity 0.80, and specificity 0.80; for 3-chamber images, the AUROC was 0.93 (95% CI 0.86 to 1.0, p < 0.001), sensitivity 0.93, and specificity 0.89; for 4-chamber images, the AUROC was 0.93 (95% CI 0.89 to 0.98, p < 0.001), sensitivity 0.84, and specificity 0.89 (Fig. 3A-C, respectively).

Finally, the classifier remained effective in identifying inadequately planned cines even in the presence of clinically relevant arrhythmia or breathing artifact (AUROC 0.83 when clinically relevant artifact was present versus 0.88 when absent; Supplementary Appendix Fig. A2).

### 3.2 Quality assessment of movement artifact in long- and short-axis images

A total of 118 cine images (33 4-chamber, 26 2-chamber, 30 3-chamber, and 29 SAX) from 101 unique studies were assigned to the testing dataset and were used to assess the AI’s ability to continuously judge image quality in terms of freedom from movement (arrhythmia or breathing) artifacts.

The AI’s assessments were compared with the expert-derived quality assessments, yielding a Spearman’s rho of 0.81 (p < 0.001) (Fig. 2D). Subgroup analyses for each view are provided in Supplementary Appendix Fig. A3 and Table A3.

Through its continuous ratings of image quality, the AI was able to function as a binary classifier through thresholding and thereby identify images that were diagnostically inadequate in terms of movement artifact and needed repeating, with an AUROC of 0.90 (95% CI 0.84 to 0.96, p < 0.001), sensitivity of 0.85, and specificity of 0.90 (Fig. 3D).

Finally, the classifier remained effective in identifying clinically relevant movement artifact in cines even in the presence of inadequate planning (AUROC 0.87 when clinically relevant artifact was present versus 0.90 when absent; Supplementary Appendix Fig. A2).

### 3.3 Inter- and intra-reader variability

Inter-reader variability may serve as an upper limit for what an AI can achieve against a consensus of experts, as disagreements between experts indicate an element of subjectivity in the ground truth. Across all four tasks, the macro averaged AUROC between the expert who performed the continuous image quality assessments versus the expert consensus was 0.93. For the AI (versus the expert consensus), this was 0.91. Intra-reader variability for the expert was 0.95. Full comparisons between each expert are shown in Supplementary Appendix Table A4.

### 3.4 Implementation

**Fig. 4:**
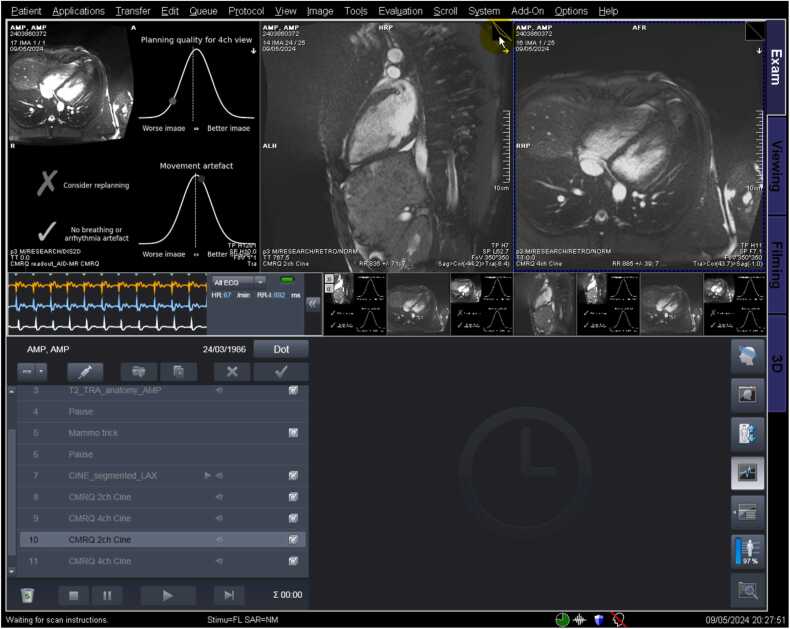
Implementation of the prototype running on a Siemens 1.5T Aera scanner in an anonymized healthy volunteer. The scanning interface (syngo E11C) on a Siemens 1.5T Aera scanner shows the results of real-time inference using the neural network. The top half of the image shows acquired images. In the top right panel, we see a misplanned 4-chamber cine, which has been positioned too low. In the top left panel, we have the results of the AI quality analysis, which is broken down into four quadrants. In the top left quadrant, we see a thumbnail of the 4-chamber cine that has been analyzed. We also see the AI’s judgment for both planning quality (top normal curve) and freedom from motion artifact (bottom normal curve). Because the image quality is below the threshold (dotted line) for planning quality, the system advises the user to consider replanning (bottom left). However, it judges the image to be sufficiently free from breathing or arrhythmia artifacts. *AI* artificial intelligence.

Fig. 4 shows the implementation of the AI at our hospital on a Siemens 1.5T Aera scanner on an anonymized healthy volunteer. The delay between the cine acquisition finishing and results of the AI analysis being visible on the scanner was 3 s. A full video of the acquisition is available in the Online Supplements.

## 4 Discussion

This study shows an AI is able to assess CMR cine imaging for quality across separate domains of planning and freedom from arrhythmia and breathing artifact. Furthermore, these quality scores can be used to identify cine images that physicians would designate as diagnostically inaccurate and should therefore be reacquired.

Previous work has investigated the ability of AI to identify CMR artifacts and has reported AUROCs for detecting artifacts in the range of 0.81 to 0.92. However, these have been limited to a single view (SAX cine imaging only) and have aimed to classify CMR cines by framing artifact detection as a binary classification task [22], [23]. In our work, we are able to produce continuous quality scores, which allow them to be contextualized.

### 4.1 Continuous quality scores can be contextualized to the clinical question

**Fig. 5:**
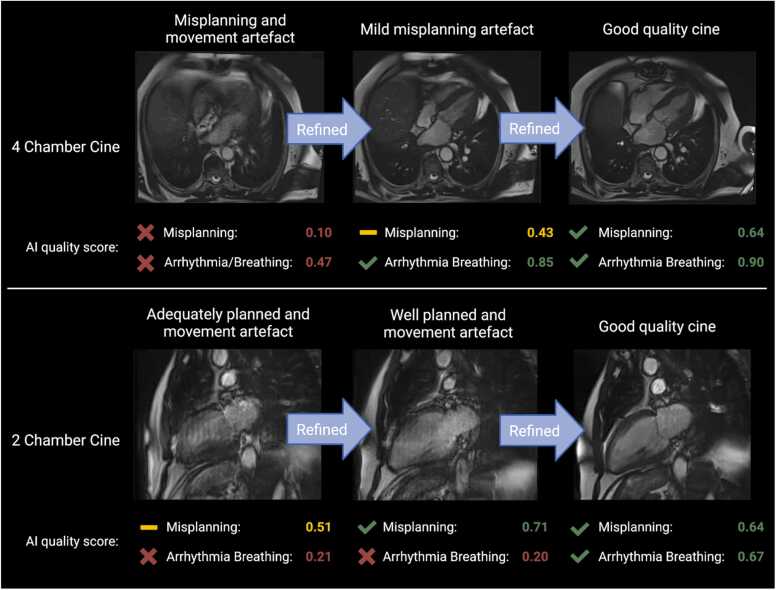
Central illustration showing two example cases where the AI identifies iterative improvements in both planning and freedom from movement artifacts. Two cases from the testing dataset were chosen where a cine was performed and was identified by the AI as suboptimal. Further two cines for each scan were performed showing incremental improvements in both slice planning and freedom from arrhythmia and/or breathing artifacts. The AI was able to identify that the radiographers had successfully resolved the quality issues, across the two quality domains. *AI* artificial intelligence.

We argue artifacts are almost invariably continuous in nature, and thresholding these into adequate/inadequate makes labeling difficult for humans and the task more difficult for the neural network at the decision boundaries. For example, one doctor may find an image adequate, while another may want it repeated. Furthermore, the quality required for an image may be specific to the patient and the type of artifact. An image that foreshortens the left ventricle in the 3-chamber view alone may be acceptable in a patient with myocarditis but may require repeat scanning in a patient referred with suspicion for apical hypertrophic cardiomyopathy. This system does not simply dichotomize studies into normal/abnormal but instead provides continuous quality scores. The prototype shown in Fig. 4 and the Supplementary Video shows how these can be compared with a threshold and lead to specific recommendations. A slightly more nuanced approach could be a “traffic light” system, where images of very high quality remain unflagged (green), intermediate quality (yellow) are prioritized for review by radiographers, and low-quality (red) result in advice for them to be repeated (Fig. 5).

### 4.2 Artifact-specific quality grading enables dynamic protocolling

This study shows AI can assess cines for image quality across distinct domains: both for the quality of planning and for movement artifacts (from breathing or arrhythmia). Using these ratings, the AI is able to accurately predict whether level 3 CMR-trained physicians would advise the image requires reacquisition. This ability to assess *why* the image needs acquisition is key: if the image is misplanned then localizers must be repeated, but in the presence of movement artifact, radiographers should focus on patient communication, enabling prospective gating, or using real-time imaging. In the event of both, a combined approach may even be required. Therefore, this AI could form the basis of a dynamic protocolling system that provides advice personalized to the patient.

## 5 Study limitations

This was a single-center study across three scanners at a single field strength (1.5T). However, it utilized scans across both GE and Siemens machines, acquired by 92 unique radiographers. This large number of operators may help to ensure this dataset still represents a range of clinical practices.

At present, our implemented solution only works on Siemens scanners, as we utilize the FIRE framework [21] for real-time image dispatching. However, this could be expanded to other vendors that provide analogous dispatching for AI inference during the acquisition process, along with the ability for processed images to be integrated into the workflow for radiographer review.

To assess the ability of the AI to identify cases that experts would identify as needing reacquisition, we asked experts to grade individuals' cines as adequate or inadequate. However, in practice, this decision is based on the clinical question and is highly reporter-dependent, with inter- and intra-reader variability evident in our results (Supplementary Appendix Table A4). However, we argue this demonstrates the utility of a system that can provide continuous quality scores, which could be tailored to the performing physician’s personal threshold and the clinical history. Furthermore, this reporter dependence underlines the importance for any gold standard labels to be derived from a consensus of experts.

In the context of this subjective gold standard, the sensitivities in this study ranged between 80% and 93%, and specificity between 80% and 90%. As with all binary classifiers, any decision threshold inherently involves a trade-off between these parameters, and our prespecified method of threshold choice (Youden’s index) weighs these equally. In practice, a higher sensitivity may be favored to ensure inadequate cases are very rarely missed. However, such an approach raises the risk of false positives, radiographers developing “alarm fatigue,” and the system becoming redundant [24]. This underlines the importance of post-implementation surveillance of any AI system.

The impact of threshold choices may be particularly important when using AI to judge freedom from movement artifact. Here, classification is performed across both LAX and SAX images and there is scope for performance to vary across views. Supplementary Appendix Fig. A3 shows AI-judged image quality in terms of movement artifact correlates less well with expert judgment for the SAX view (Spearman’s rho 0.65) than LAX views (0.81 to 0.86)—although reassuringly the area under the curve remains high at 0.88, with 100% sensitivity and 70% specificity (Supplementary Appendix Table A3).

A minority (1.9%) of cases were performed for the evaluation of congenital disease. This therefore raises questions about the generalizability of the model to patients with highly atypical cardiac anatomy. Further work will need to be undertaken to assess and likely improve the performance in such cases.

## 6 Conclusion

AI can independently assess distinct key domains of LAX and SAX CMR cine quality. It can quantify both planning quality and the freedom from arrhythmia or breathing artifacts. These ratings can be used to identify cases where acquisition is warranted and could guide specific corrective actions to optimize image quality, such as replanning, prospective gating, or real-time imaging.

## Funding

J.P.H. is funded by the 10.13039/501100000274British Heart Foundation (FS/ICRF/22/26039). This project was partly funded by the 10.13039/501100000265Medical Research Council (MR/X502959/1) and the 10.13039/501100000833Rosetrees Trust (IAA 2023\1) via the Imperial College Impact Acceleration Award.

## Author contributions

J.P.H. conceived the study. J.P.H. and H.C.C. designed the study. J.P.H., G.D.C., K.V., and S.Z. analyzed the data. J.P.H., M.J.S., and H.C.C. performed statistical analysis. J.P.H. and H.C.C. drafted the initial manuscript. J.P.H., H.C.C., M.J.S., D.P.F., G.D.C., K.V., S.Z., and M.M. revised the manuscript.

## Ethics approval and consent

Ethical approval was granted by the Health Research Authority (Integrated Research Application System number 309103). The requirement for individual patient consent was waived.

## Consent for publication

Not applicable.

## Declaration of competing interests

The authors declare the following financial interests/personal relationships which may be considered as potential competing interests: Dr. James Howard reports financial support was provided by British Heart Foundation, Medical Research Council, and Rosetrees Trust. Dr. James Howard and Dr. Graham Cole report a relationship with Mycardium AI Limited that includes equity or stocks. The other authors declare that they have no known competing financial interests or personal relationships that could have appeared to influence the work reported in this paper.

## Data Availability

The testing datasets used and/or analyzed during the current study are available from the corresponding author on reasonable request. Code and trained models may not be shareable under the terms of Dr. Howard’s funding with the British Heart Foundation.
