# Supplementary material for: Automating quality control in cardiac magnetic resonance: Artificial intelligence for discriminative assessment of planning and motion artifacts and real-time reacquisition guidance
Source: J Cardiovasc Magn Reson. 2024 Jul 28;26(2):101067. doi: 10.1016/j.jocmr.2024.101067 (PMC11416635; doi:10.1016/j.jocmr.2024.101067)
Supplement: Supplementary file 1 — Supplementary material [file mmc1.docx]

Supplementary Appendix

**Figure A1.** The impact of the selective back propagation using pseudolabels for non-present views on AI-judged misplanning.


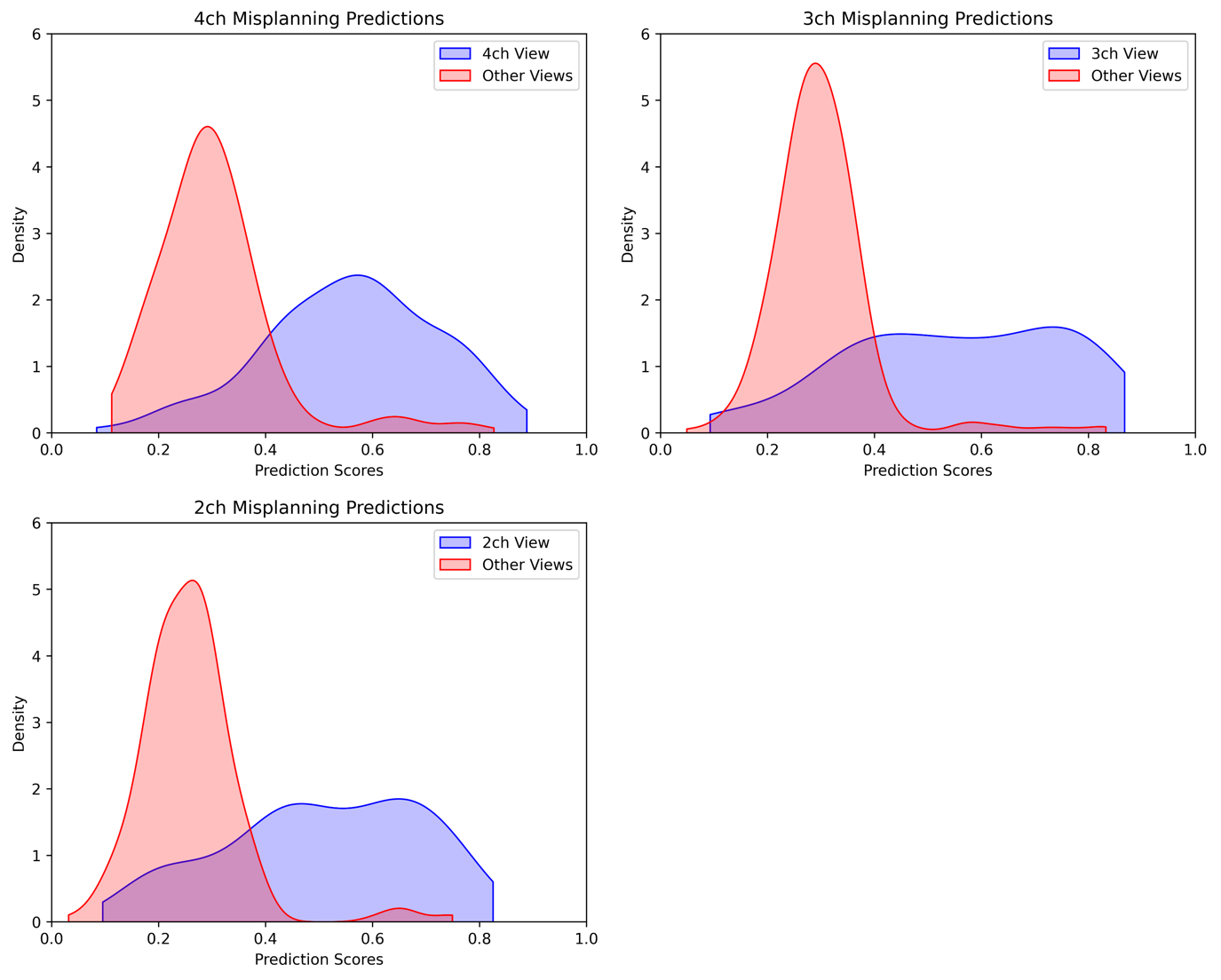


**Figure A1.** Distributions of AI-judged misplanning. In blue we quality scores for the intended view (e.g. in the top left plot, how well planned 4-chamber videos are compared to the ideal 4-chamber video). In red, we see the distribution of quality scores for the other views (e.g. in the top left plot, how well planned 2- and 3-chamber videos are compared to the ideal 4-chamber view). The reason these are not clustered around 0 is because the most misplanned images contain no heart altogether; a well-planned 4-chamber image still contains some features of a good 2-chamber image such as the left atrium and a non-foreshortened apex. Our selective back propagation approach, discussed in the Methods, explains how these distributions were imposed.

**Table A1.** Comparison of multiple single networks versus a combined network approach.

|  | Performance (Spearman’s rho) | |
| --- | --- | --- |
| Task | Single Networks | Combined Network |
| 4ch Misplanning | 0.78 | 0.81 |
| 3ch Misplanning | 0.83 | 0.84 |
| 2ch Misplanning | 0.77 | 0.84 |
| Arrhythmia or Breathing Cines (2/3/4 chamber and short-axis) | 0.80 | 0.81 |

**Table A2.** Clinical indications for studies.

| Indication | Number of studies  (% of total; N = 1142) |
| --- | --- |
| Cardiomyopathy | 311 (27.2%) |
| Stress | 176 (15.4%) |
| Function and viability | 174 (15.2%) |
| Myocarditis/acute myocardial injury | 129 (11.3%) |
| Hypertrophic cardiomyopathy | 89 (7.8%) |
| Complex aorta | 83 (7.3%) |
| Complex valvular | 59 (5.2%) |
| Pulmonary hypertension | 58 (5.1%) |
| Congenital heart disease | 22 (1.9%) |
| Iron load | 13 (1.4%) |
| Cardiac masses | 9 (0.8%) |
| Pericardial disease | 9 (0.8%) |
| Research | 8 (0.7%) |
| Function (non-contrast) | 2 (0.2%) |

**Figure A2.** Ability of AI to identify cines judged clinically inadequate and requiring repeating on the testing set, broken down by the absence/presence of other artefacts.

**
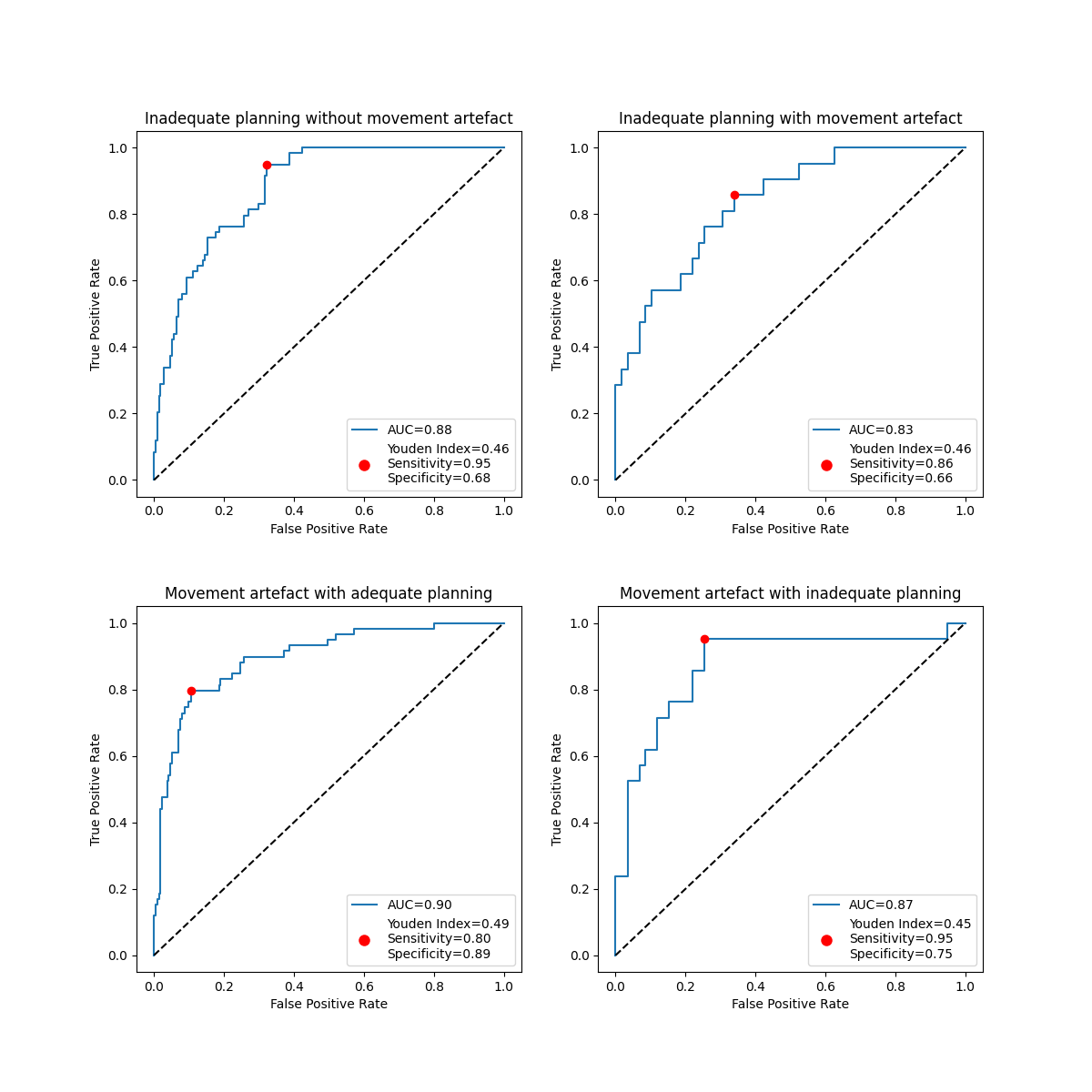
Figure A2.** Receiver Operating Characteristics curves for the AI in identifying cines that are clinically inadequate and therefore require repeating. Youden Indices and corresponding sensitivities and specificities are also illustrated.

**Figure A3**. Correlation between AI-judged and expert-judged image quality on the testing set in terms of freedom from movement (arrhythmia or breathing) artefact.


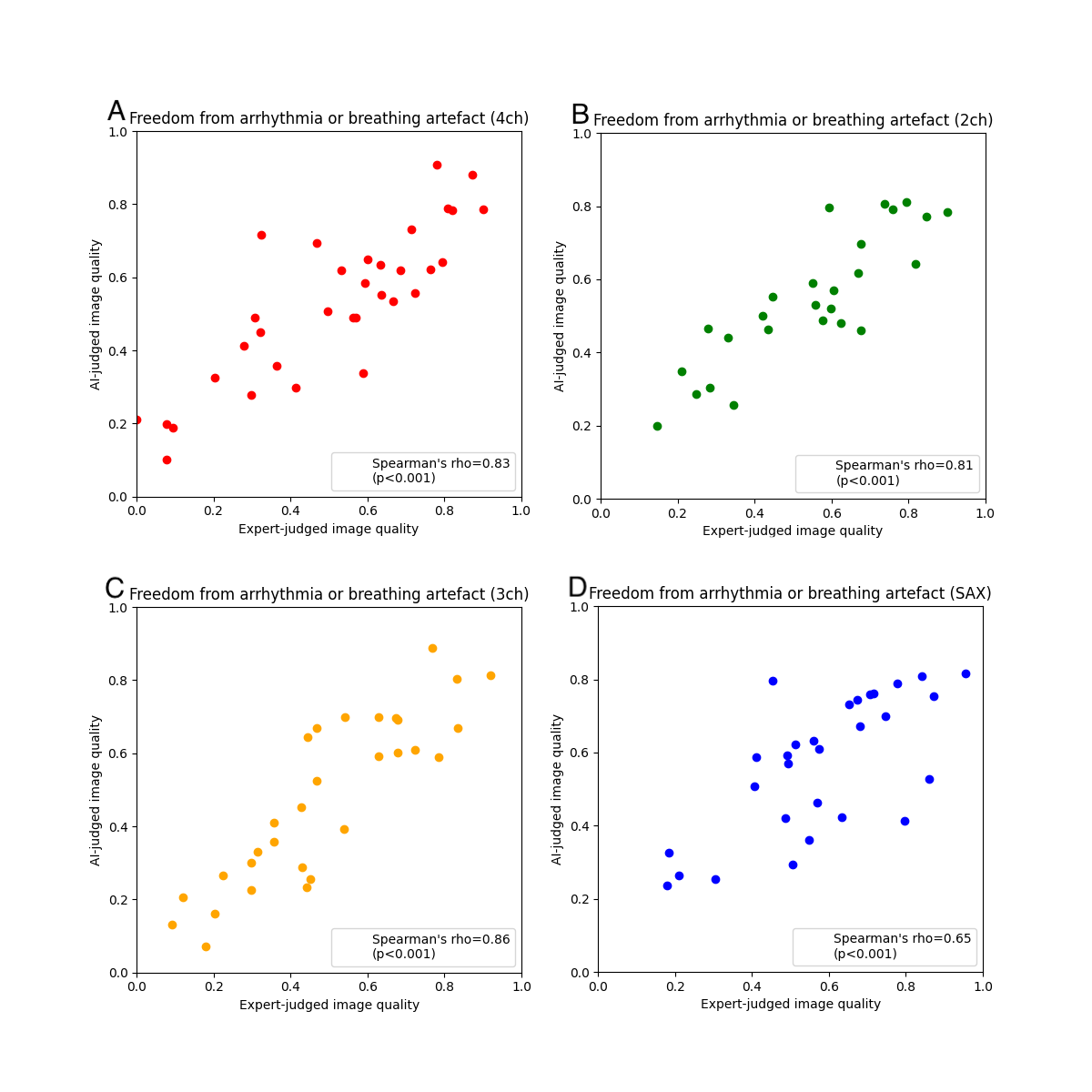


**Figure A3.** Spearman’s rank correlation between AI and expert assessments of freedom from movement artefact (A: Freedom from movement artefact in 4-chamber cines; B: Freedom from movement artefact in 2-chamber cines; C: Freedom from movement artefact in 3-chamber cines; D: Freedom from movement artefact in short-axis cines).

**Table A3.** Classification performance for the AI in identifying cines that are clinically inadequate and therefore require repeating, analysed by view.

|  | 4ch | 3ch | 2ch | SAX |
| --- | --- | --- | --- | --- |
| AUC | 0.90 | 0.93 | 0.92 | 0.88 |
| Youden Index | 0.51 | 0.55 | 0.51 | 0.41 |
| Sensitivity | 0.80 | 1.00 | 0.91 | 1.00 |
| Specificity | 1.00 | 0.88 | 0.93 | 0.70 |

**Table A4.** Inter-reader variability results showing macro-averaged AUROCs over the 4 tasks. These values represent the continuous quality assessments made by the expert (left column) and AI (right column) in identifying clinically inadequate images as judged by experts (rows). The inter-reader variability between an expert and the consensus had a corresponding AUROC of 0.93, and the intra-reader variability of 0.95.

* To allow inter-expert variability to be measured, Expert’s 1s clinical opinion is not used to calculate the expert consensus in this Table.

|  | Predictor | |
| --- | --- | --- |
| Gold standard | Expert 1 | AI |
| Expert 2 (External) | 0.91 | 0.90 |
| Expert 3 (External) | 0.92 | 0.89 |
| Expert 4 (External) | 0.86 | 0.85 |
| *Expert 1 (Internal*)* | *0.95* | *0.89* |
| Expert consensus* | 0.93 | 0.91 |
