# Supplementary figures and images for: Automating quality control in cardiac magnetic resonance: Artificial intelligence for discriminative assessment of planning and motion artifacts and real-time reacquisition guidance
Source: J Cardiovasc Magn Reson. 2024 Jul 28;26(2):101067. doi: 10.1016/j.jocmr.2024.101067 (PMC11416635; doi:10.1016/j.jocmr.2024.101067)

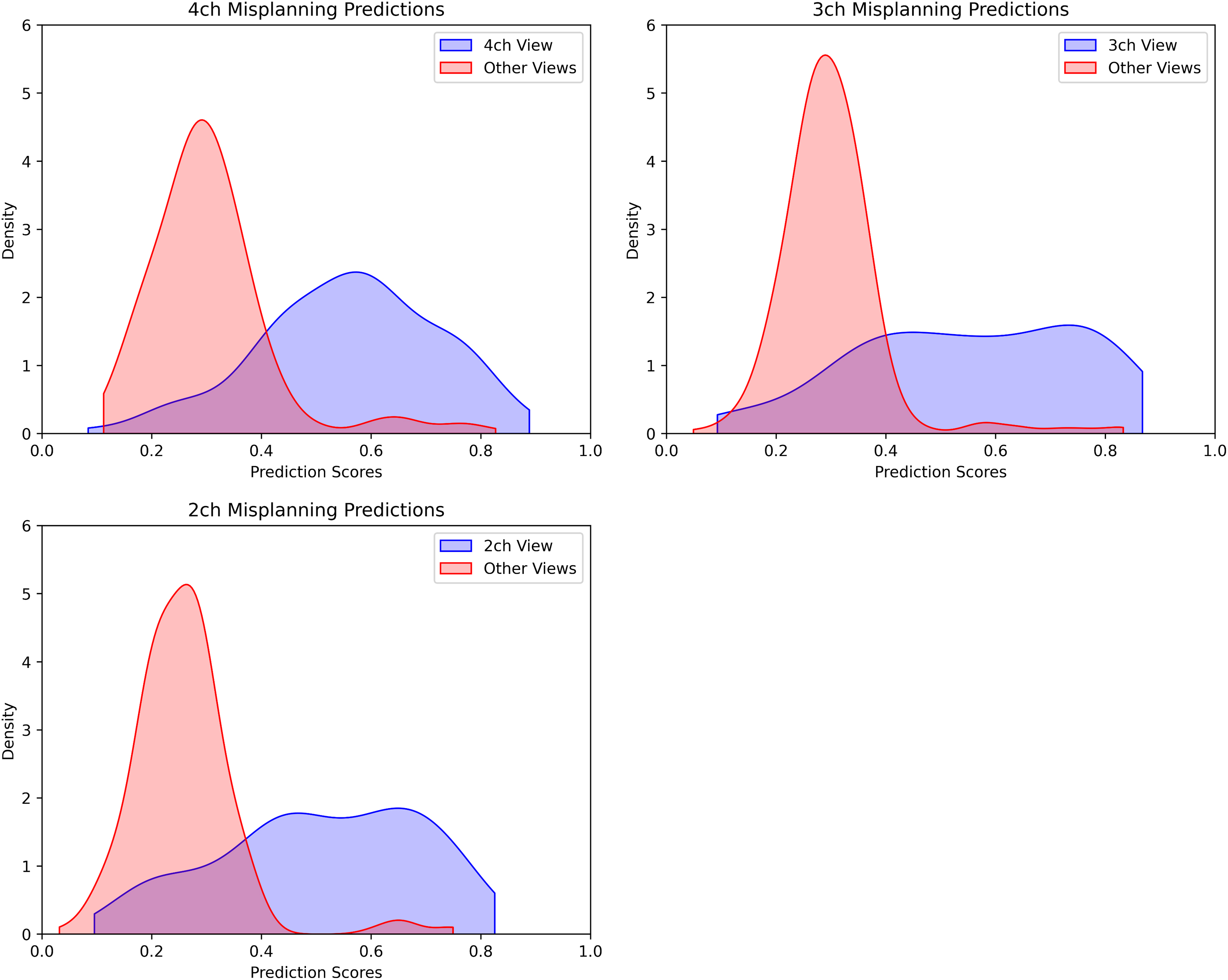

Supplement: Supplementary file 3 — Supplementary material [file mmc3.jpg]

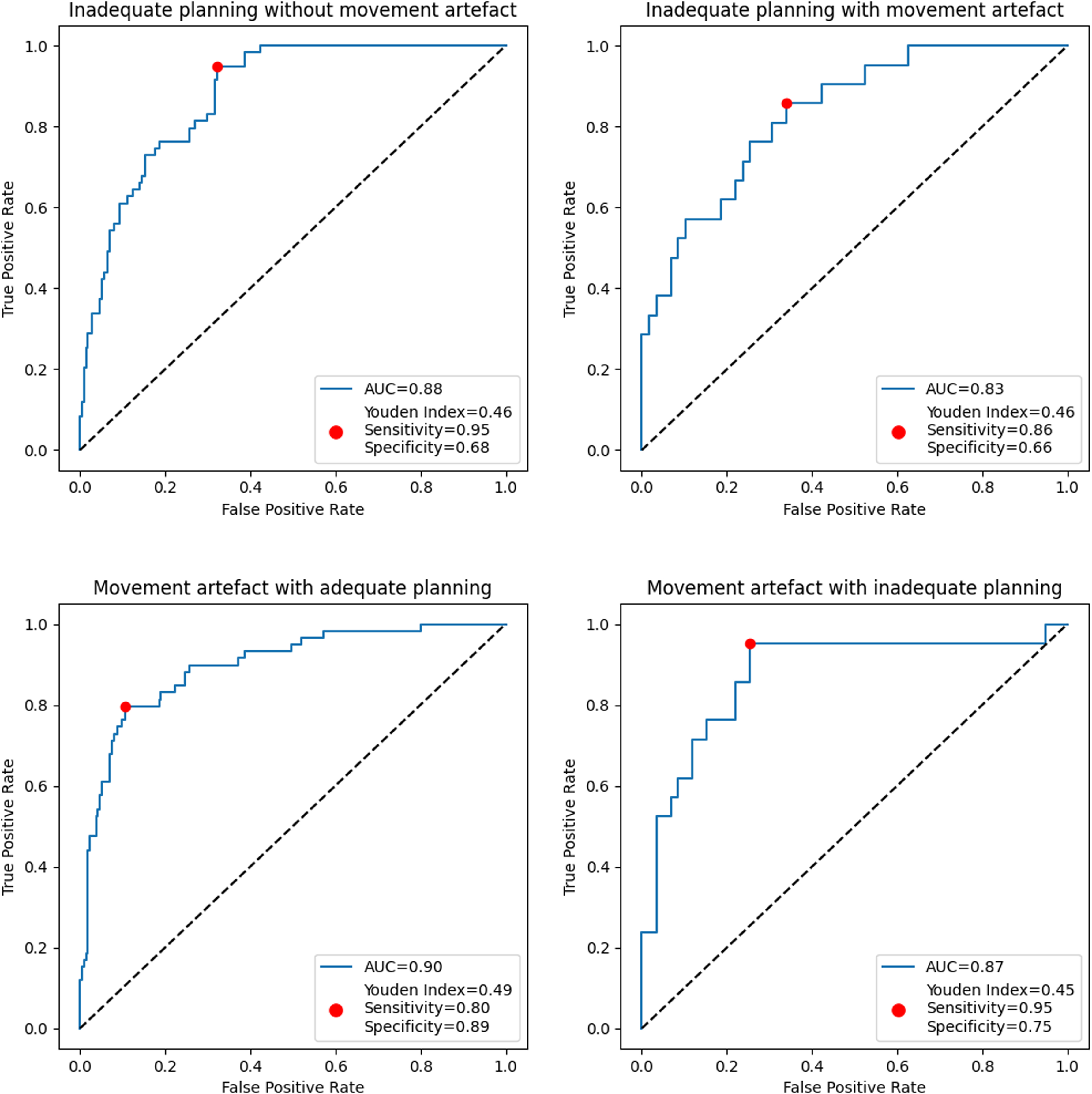

Supplement: Supplementary file 4 — Supplementary material [file mmc4.jpg]

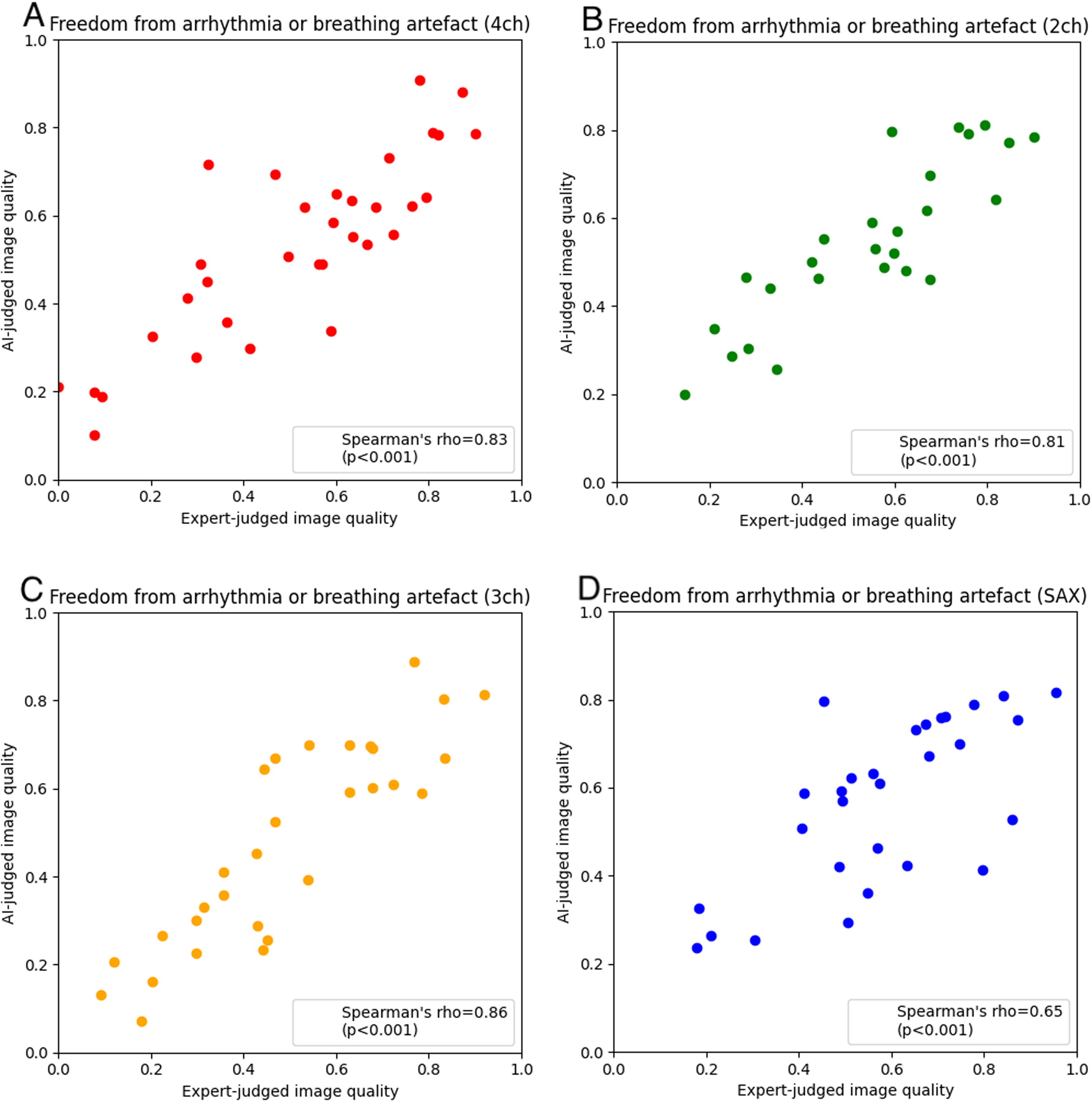

Supplement: Supplementary file 5 — Supplementary material [file mmc5.jpg]
